# Polymorphisms in the *MASP1* Gene Are Associated with Serum Levels of MASP-1, MASP-3, and MAp44

**DOI:** 10.1371/journal.pone.0073317

**Published:** 2013-09-02

**Authors:** Christian Gytz Ammitzbøll, Rudi Steffensen, Hans Jørgen Nielsen, Steffen Thiel, Kristian Stengaard-Pedersen, Martin Bøgsted, Jens Christian Jensenius

**Affiliations:** 1 Department of Rheumatology, Aarhus University Hospital, Aarhus, Denmark; 2 Department of Clinical Immunology, Aalborg University Hospital, Aalborg, Denmark; 3 Department of Surgical Gastroenterology, Hvidovre University Hospital, Hvidovre, Denmark; 4 Department of Biomedicine, Aarhus University, Aarhus, Denmark; 5 Department of Haematology, Aalborg University Hospital, Aarhus, Denmark; 6 Department of Mathematical Sciences, Aalborg University, Aarhus, Denmark; University of California Merced, United States of America

## Abstract

**Introduction:**

MASP-1 is the first protein in the activation of the lectin pathway and MASP-1 is, like its isoforms MASP-3 and MAp44, encoded by the *MASP1* gene. Our aim was to explore associations between polymorphisms in *MASP1* and corresponding concentrations of MASP-1, MASP-3, and MAp44 in plasma as well as the genetic contribution to the equilibrium between the three proteins.

**Methods:**

Fifteen SNPs were genotyped in the *MASP1* gene in 350 blood donors. Corresponding plasma concentrations of MASP-1, MASP-3, and MAp44 were measured.

**Results:**

A total of 10 different SNPs showed associations with the concentration of one or some of the three proteins (rs113938200, rs190590338, rs35089177, rs3774275, rs67143992, rs698090, rs72549154, rs72549254, rs75284004, rs7625133), and several of these were in strong linkage. SNPs located in the mutually exclusive splice region had opposite effects on the protein concentrations. Being e.g. homozygote for the minor allele of rs3774275 was associated with an increase in median concentration of 13% in MASP-1(P=0.03), 29% in MAp44 (P<0.001), and a decrease in MASP-3 of 26% (P<0.001) compared to homozygosis for the major allele. Heterozygosis of rs113938200 (*p.Asn368Asp* in MAp44) was associated with a reduced MAp44 concentration of 61% (P=0.005). Rs190590338 located in the promoter region was associated in the heterozygote form with an increased MASP-1 concentration of 35% (P = 0.002). A multivariate linear regression model including sex, age, M- and H-ficolin, MBL, and the 15 SNPs explained 20-48% of the variation in the concentration of the three proteins and the SNPs investigated contributed with the most explanatory power (12-23%).

**Discussion:**

The present study described 10 SNPs, which were associated with the concentration of one or some of the three proteins originating from the *MASP1* gene and in a multivariate model it was shown that the SNPs contributed with the most explanatory power to the protein concentrations.

## Introduction

The immune system has evolved innate and adaptive components that cooperate to protect against microbial infections while maintaining homeostasis of the body. The innate system encompasses various recognition molecules able to sense both exogenous and endogenous danger signals arising from pathogens or damaged host cells. The complement system is an important part of the innate immune system, consisting of a finely equilibrated composition of proteins. In order to enable the interpretation of a genotype-phenotype relationship it is relevant to study the influence of polymorphisms in the genes encoding these proteins.

The lectin pathway activates the complement system through the recognition of pathogens or altered-self-structures by mannan-binding lectin (MBL) or one of the three ficolins (H-, L-, and M-ﬁcolin). The ficolins or MBL form complexes with five structurally related proteins, the three MBL-associated serine proteases (MASPs), MASP-1, MASP-2, and MASP-3, and two non-enzymatic splice products, the MBL-associated proteins MAp19 and MAp44. Upon binding of MBL or ficolins to pathogens, MASPs molecules are converted from pro-enzymes to active forms, leading to cleavage of C4 and C2 and generation of the C3 convertase. Over the past decade new knowledge has broadened our understanding of the role of the lectin pathway from complement activation to include coagulation, autoimmunity, ischemia-reperfusion injury and embryogenesis [1–3].

**Figure 1:**
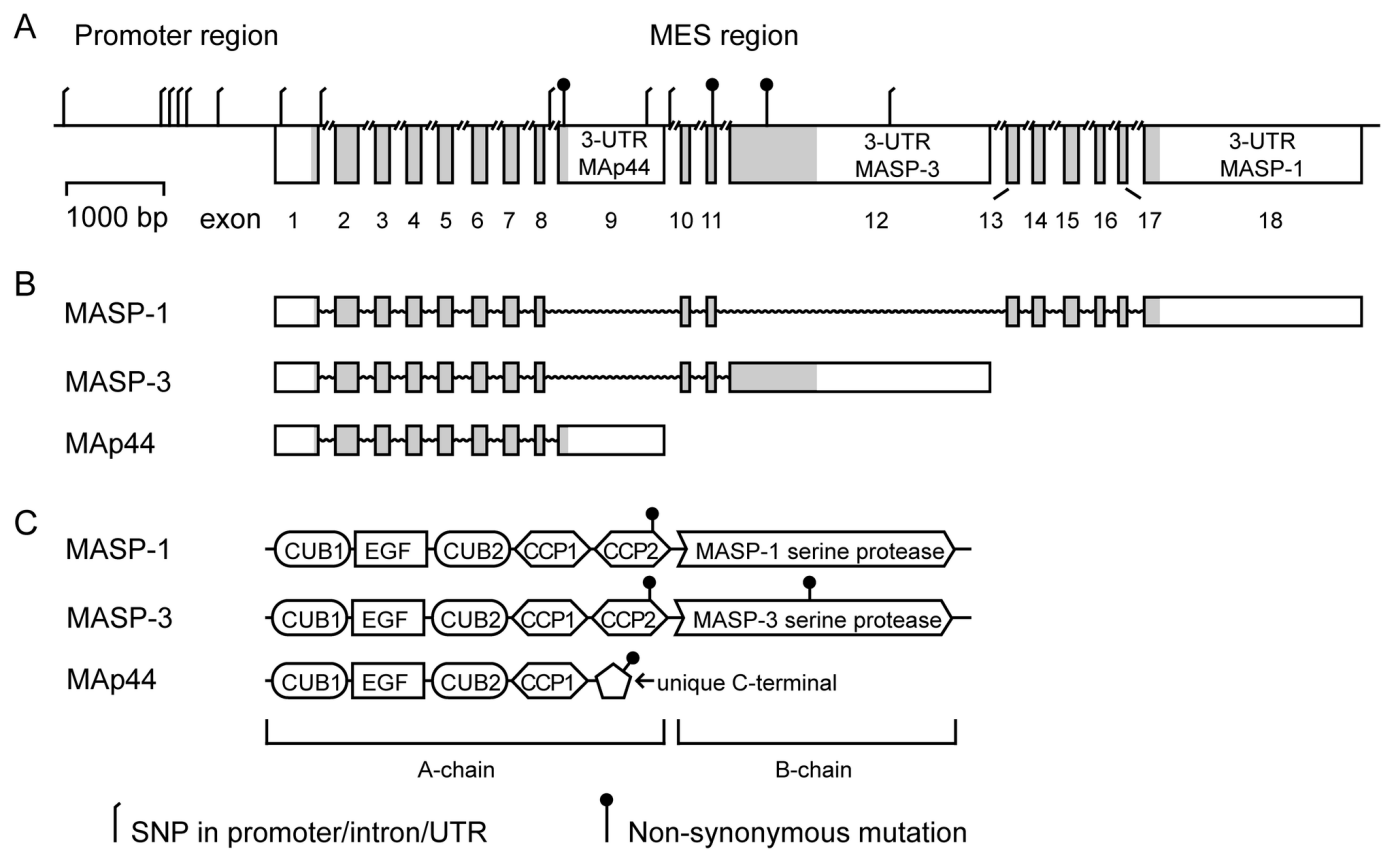
Schematic presentation of the *MASP1* gene and transcripts. **A** While the size of exons and the promoter region are drawn to scale the introns are truncated. The MES region indicates the region where the mutually exclusive splicing takes place that generates the three proteins. All SNPs investigated in this report are marked as either a non-synonymous mutation or not. Grey boxes indicated translated part of the exons. **B** The primary transcript can be spliced to three different mRNAs coding for MASP-1, MASP-3, and MAp44. **C** MASP-1 and -3 share five domains encoded by exons 1–8, 10 and 11 (the A-chain). CUB = C1r/C1s, Uegf, Bmp1; EGF = epidermal growth factor and CCP = complement control protein. They have unique serine protease domains (B-chains), encoded by exon 12 (MASP-3) and exons 13–18 (MASP-1). MAp44 shares the first four domains with MASP-1 and MASP-3, followed by a unique C-terminal, encoded by exon 9.

The *MASP1* gene, located on chromosome 3q27-28 spanning 76 kb, encodes a primary transcript, which are spliced by mutually exclusive splicing into three different mRNAs coding for MASP-1, MASP-3, and MAp44, (Figure 1) [4]. This splicing takes place in the mutually exclusive splice (MES) region which is located in between exon 8 to 13. MASP-1 and -3 share five domains encoded by exons 1–8, 10, and 11 (the A-chain). They have unique serine protease domains (B-chains), encoded by exon 12 (MASP-3) and exons 13–18 (MASP-1). MAp44 shares the first four domains with MASP-1 and MASP-3, followed by a unique C-terminal, encoded by exon 9 [4]. While MASP-1 expression is largely confined to the liver [4], MASP-3 is largely expressed in the liver, bladder, brain, cervix, colon, and prostate, and MAp44 primarily in the heart [4]. Others have reported that MAp44 (termed MAP1) may also be synthesized by skeletal muscle cells [5].

Recent discoveries have indicated that MASP-1 is critically involved in the lectin pathway, as deficiency of MASP-1 causes a functional block of the lectin pathway by lack of MASP-2 activation [6]. Activated MASP-2 activates complement factors C2 and C4 generating complement activation. MASP-1 thus appears to be required for significant activation of MASP-2 under physiological conditions [6–8]. MASP-1 exhibits thrombin-like activity by, cleaving two substrates of thrombin (fibrinogen and factor XIII), and is inhibited by antithrombin in presence of heparin. Animal studies have suggested a role of MASP-1 in the activation of the alternative pathway by activating factor D [9,10], but a recent study indicates that MASP-1 have no such role in a human setting [6]. Very little is so far known about the functional roles of MASP-3 and MAp44. Rare mutations in the MASP-3 encoding part of the *MASP1* gene have been directly linked to the 3MC syndrome, an autosomal recessive congenital syndrome, with features of facial dysmorphic traits, cleft lip and palate, post-natal growth deficiency, cognitive impairment, and hearing loss [11,12].

Single nucleotide polymorphisms (SNPs) in the genes of several of the lectin pathway proteins have been found to influence the corresponding concentrations in plasma [13–17]. So far no one has demonstrated equivalent associations concerning the 3 proteins originating from the *MASP1* gene.

Our aim in the present work was to explore correlations between SNPs in *MASP1* and concentrations in plasma of MASP-1, MASP-3, and MAp44. We first examined for new SNPs by sequencing sections of *MASP1* in 46 selected cases with very low or high protein concentrations. Afterwards we analyzed 15 SNPs in *MASP1* in 350 blood donors and evaluated for associations to the corresponding protein levels.

## Materials and Methods

### Ethics Statement

This study was approved by “The Committees on Biomedical Research Ethics of the Capital Region”. Written informed consent was obtained from all 350 blood donors that participated, and all clinical investigations were conducted according to the principles expressed in the Declaration of Helsinki.

### Subject and Samples

A cohort of 350 Danish blood donors aged 18-64 years was analyzed. Genomic DNA from peripheral blood leukocytes was extracted using the QIAamp DNA Mini Kit (Qiagen, Valencia, CA). Successful DNA extraction failed for 4 donors.

### Protein measurements

The concentrations of MASP-1, MASP-3, and MAp44 in the sera from some of these patients have been published previously [4,18,19], and we extended this to encompass the whole cohort of 350 donors.

MAp44 and MASP-3 concentrations were determined by a time-resolved immunofluorometric assay, previously described in detail [18], according to the same principle as the traditional enzyme-linked immunosorbent assay. In brief the assay is carried out as follows: diluted samples are incubated in monoclonal antibody coated microtiter wells. Next, the bound protein is detected by biotin-labeled monoclonal antibody followed by europium-labeled streptavidin and measurement of the bound europium by time-resolved fluorometry.

While the assays for MASP-3 and MAp44 are of the conventional sandwich configuration, the assay for MASP-1 is an inhibition assay, where MASP-1 in the sample in a dose–response manner inhibits the binding of anti-MASP-1 to a MASP-1 fragment coated onto the wells. The assay is previously described in detail [19]. In brief, microtiter wells were coated with recombinant MASP-1 (CCP1-CCP2-SP). Samples composed of equal volume of diluted test sample and diluted rat anti-MASP-1 antibody were incubated for 15 min to ensure the binding of anti-MASP-1 antibody to MASP-1 in the sample before adding the mixture to the wells. Following incubation with biotinylated rabbit anti-rat-Ig the wells were washed, incubated with europium-labeled streptavidin, and bound europium was measured by time-resolved fluorometry. For quality control three internal controls are added to each assay plate in all three assays.

### Identification of *MASP1* polymorphisms

Genomic DNA from the individuals with the highest and lowest concentrations of MASP-1, MASP-3 and MAp44 was chosen for SNP exploration by DNA sequencing, in total 46 individuals. Two kb of the promoter region and exon 1 and 2 of *MASP1* were sequenced. Sequencing was performed by Beckman Coulter Genomics, Danvers,, USA. The design of PCR amplicons utilized the following criteria; a 50 bp overlap where amplicons overlapped, and at intron/exon boundaries a minimum of 50 bp of intron sequence is represented and masks dbSNP polymorphisms to avoid placing primer on SNP containing regions. A test PCR reaction at a standard thermal cycling condition was performed on each amplicon using control DNA specimens, followed by sequencing. High-throughput PCR setup and sequencing included the following steps: PCR reaction setup into 384 well format plates and thermal cycling, PCR purification utilizing SPRI (solid-phase reversible immobilization), bi-directional DNA sequencing using BigDye Terminator v 3.1, post reaction dye terminator removal using Agencourt CleanSEQ and sequence delineation on an ABI PRISM 3730xl with base calling and data compilation. Sequence data generated from samples were assembled along with a reference sequence, and afterwards automated polymorphism detection using Polyphred. The SNPs not encountered in the dbSNP database were submitted to NCBI Reference Assembly and reported here with an ss-number.

### Genotyping

The TaqMan OpenArray genotyping system from Applied Biosystems (ABI, Foster City, CA, USA), which is a high-throughput, highly automated and relatively low-cost (per assay) system that allow testing of many SNPs in multiple individuals in parallel, was used for genotyping of 14 SNPs in the *MASP1* gene. We typed 10 SNPs with custom-designed genotyping assays and four SNPs with predesigned TaqMan SNPs assays (see table S1 for assay information). OpenArray plates were manufactured by Applied Biosystems. DNA samples were diluted to a final concentration of 50 ng/µl in 96-well plates. Nontemplate control blanks were randomly distributed on all 96-well plates in order to estimate the quality of the samples. 384-well plates with DNA were prepared by a Biomek NX (Beckman Coulter, Fullerton, USA). 100 ng of DNA was mixed with 2 µl TaqMan Open Array mastermix (ABI, Foster City, CA, USA) in 384-well plates, loaded on the Open Array plates using the OpenArray NT Autoloader. Polymerase chain reaction was performed using GeneAmp 9700 thermal cycler and amplification were performed according to the following: 91 °C × 10 min + 50(51 °C × 23 s + 53.5 °C × 30 s + 54.5 °C × 13 s + 97 °C × 22 s + 92 °C × 7 s) 20 °C × 5 min + 4 °C ∞. Fluorescence of the FAM and VIC reporters was read with the OpenArray® NT Imager. Data were analyzed with the Biotrove® OpenArray® SNP Genotyping Analysis Software package version 1.0.3.

One SNP (rs72549254) was performed as single custom-designed TaqMan assay since assay design for Open Array failed. DNA amplification was carried out in 5-µl volume containing 20 ng DNA, 0.9 µm primers and 0.2 µm probes (final concentrations), amplified in 384-well plates. PCRs were performed with the following protocol on a GeneAmp PCR 9700 (Applied Biosystems): 95 °C for 10 min, followed by 40 cycles of 95 °C for 15 s and 60 °C for 1 min. Subsequently, end-point fluorescence was determined using the ABI PRISM 7900 HT Sequence Detection Systems and the SDS version 2.3 software (ABI, Foster City, CA, USA).

### Statistical analysis

The Haploview software 4.2 [20] was used to test the genotype distributions for deviation from Hardy-Weinberg equilibrium and estimate the degree of linkage disequilibrium (LD) between the SNPs. D’ is a measure of the amount of LD between two genetic loci. A value of 0 indicates that the two loci are in complete equilibrium (independent of one another), whereas 1 represents 100% linkage (the highest amount of disequilibrium possible is present). The LOD score (logarithm (base 10) of odds) compares the likelihood of obtaining the test data if the two loci are indeed linked to the likelihood of observing the same data purely by chance. High LOD scores favor the presence of linkage, and a LOD score of +2 indicates 100 to 1 odds that the linkage being observed did not occur by chance. The squared Pearson’s correlation coefficient (R^2^) was used as another measure of LD between pairs of SNPs.

Statistical analysis was performed using the statistical software system R, version 2.15.3 [21]. Student’s t-test was used to test population differences for continuous variables and Pearson’s Chi-square was used to test for population differences for categorical variables. Protein concentrations in serum were log-normally distributed and, therefore, log-transformed (natural logarithm) before analysis. Analysis of variance (ANOVA) based on multiple linear regression models was used to investigate the association between the outcome variable protein concentration in serum and the covariates age, gender, genotypes, and the concentration of MBL, H-, and M-ficolin. The adequacy of the multiple linear regression models were controlled by qqplots of the residuals, results not shown.

The Haploview software 4.2 [20] was used to infer haplotypes on a group basis by the ‘confidence interval’ method using default settings. Tests of haplotype association with protein levels were performed by the ‘haplo.stats’ R-package, version 1.6.3 [22]. For all analyses we assume additive haplotype effects and Gaussian distributed traits/phenotypes. First ‘haplo.score’ was used to study association between the combined haplotypes and levels of each of the three MASP1 related proteins, by calculating global P-values. Next, the ‘haplo.glm’ function was used to estimate the effect of each haplotype compared to the most frequent haplotype (H1). In contrast to assigning the most likely haplotype phase resolution to each sample the ‘haplo.glm’ function estimates a generalized linear model by incorporating the haplotype phase uncertainty by inferring a probability matrix of haplotype likelihoods for each individual by use of the expectation-maximization (EM) haplotype-inference algorithm.

A simple way to measure the proportions of variance explained in an ANOVA based on a multiple linear regression model is to divide the sum of squares for each covariate by the total sum of squares. These ratios represent the proportion of variance explained for each covariate. The proportion of the variation for each covariate age, gender, genotypes, MBL, H- and M-ficolin, and the unexplained variation in the multiple linear regression models was calculated and depicted by pie charts.

The residuals of a single multivariate regression model with the protein concentrations of MASP-1, MASP-3, and Map44 as outcome variables and the covariates age, gender, genotypes, and the concentration of MBL, H-, and M-ficolin was calculated. The remaining degree of association between two of the three proteins, while keeping the third constant, was measured by partial correlations of the residuals. Throughout effects are reported as predicted geometric mean concentrations and 95% confidence intervals are used. Results with P-values below 0.05 were considered significant.

## Results

### Effect of age and gender on concentration

Blood donor characteristics showed a majority of men and a median age of 47 years (Table 1). Prior to the SNP association analysis, the association of age and gender and their combined association with the serum concentration of the three proteins were tested using multiple linear regression models, with serum concentration as outcome variable and age and gender as covariates.

**Table 1:** Blood donor characteristics.

Male/female, number	218/132	(62%/38%)
Median age (IQR)	47	(39-55)
MASP-1, µg/ml (CI)	9.60	(9.29-9.92)
MASP-3, µg/ml (CI)	5.00	(4.83-5.16)
MAp44, µg/ml (CI)	1.74	(1.69-1.78)

Concentrations are medians calculated from log-transformed data. IQR = inter quartile range. CI = 95% confidence intervals.

**Figure 2:**
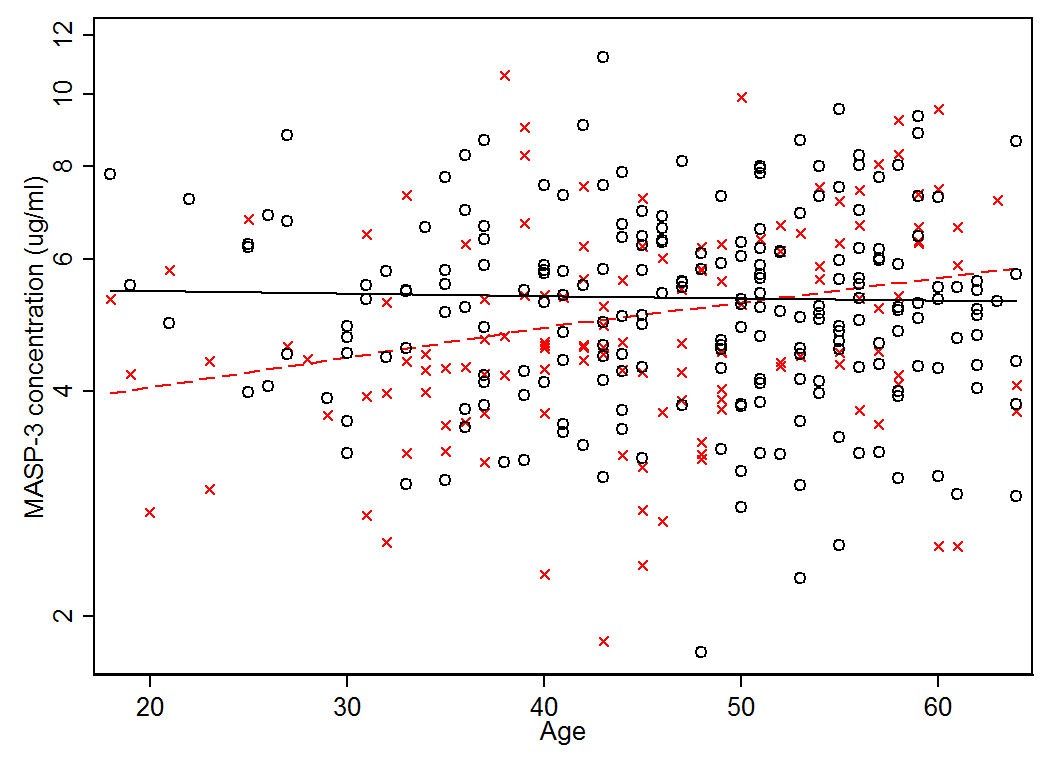
The relationship between age, gender, and MASP-3 concentration. X = female, O = male. The dashed line indicated the best fitted line for females and the solid line for males.

A significant association of the serum concentration of MASP-3 with gender and age (P = 0.003, both) and with an effect of interaction between age-gender was observed (P = 0.008). As an example this means that a 40 year old man would have 11% more MASP-3 than a 40 year old woman, while a 60 year old woman has a 6% higher MASP-3 concentration than a similar aged man (Figure 2). No effects of either age, gender or their interaction were observed for MASP-1(P > 0.12) and MAp44 (P > 0.57).

### SNP Exploration of *MASP1*

By sequencing the promoter region, exon one, and exon two of the *MASP1* gene in 46 individuals we discovered 19 SNPs of which 9 at the time of discovery were not registered with a rs-number in the dbSNP Build 133 database at the NCBI Reference Assembly (table S2). Nine SNPs were located in the promoter region, six in the 5´-UTR region, and 4 in introns, while none were found in exons.

### Genotypes and concentrations

Based on the above findings and the SNPs listed in the dbSNP Build 133 database at the NCBI Reference Assembly, 15 SNPs were chosen to be genotyped in 346 blood donors (Table 2). The SNPs selected were located in two different regions of the gene, in the promoter and MES region, as we speculated polymorphisms in these two regions would be the most proposing to investigate with respect to effect on phenotype. No deviations from Hardy-Weinberg equilibrium were found for any of the genotyped SNPs (results not shown). To test for the association between genotypes and serum concentrations of MASP-1, MASP-3, and MAp44 a multiple linear regression model with serum concentration as outcome variable and all genotypes as independent variables was applied. Highly significant associations were found between all genotypes and serum concentration of MASP-1(P = 0.006), MASP-3(P < 0.0001), and MAp44 (P < 0.0001).

**Table 2:** Effect of 15 SNPs in *MASP1* on the concentration of MASP-1, MASP-3, and MAp44.

**Pos.**	**rs-nr**	**Gen.**	**n**	**MASP-1**			**MASP-3**			**MAp44**		
	- region			**Concentration**	**Δ%**	**p**	**Concentration**	**Δ%**	**p**	**Concentration**	**Δ%**	**p**
-2464	**rs190590338**	GG	330	9.48 (9.17;9.80)			5.00 (4.83;5.17)			1.73 (1.69;1.78)		
	- promoter	GA	11	12.82 (10.67;15.40)	35%	0.002	4.75 (3.95;5.72)	-5%	0.600	1.84 (1.59;2.12)	6%	0.433
-1495	**rs143668135**	C C	332	9.60 (9.28;9.93)			4.98 (4.81;5.15)			1.74 (1.69;1.79)		
	- promoter	C T	10	9.10 (7.49;11.06)	-5%	0.598	5.30 (4.36;6.43)	6%	0.533	1.51 (1.29;1.76)	-13%	0.071
-1479	**rs75284004**	AA	320	9.59 (9.27;9.92)			5.05 (4.88;5.22)			1.72 (1.68;1.77)		
	- promoter	AG	22	9.83 (8.63;11.20)	2%	0.720	4.22 (3.71;4.80)	-16%	0.009	1.89 (1.70;2.09)	10%	0.089
-1418	**rs35089177**	TT	164	10.20 (9.73;10.69)			4.87 (4.64;5.10)			1.81 (1.74;1.87)		
	- promoter	TA	143	9.14 (8.69;9.61)	-10%	0.002	5.07 (4.82;5.34)	4%	0.242	1.70 (1.63;1.77)	-6%	0.025
		AA	36	8.69 (7.86;9.61)	-15%	0.005	5.22 (4.71;5.78)	7%	0.223	1.55 (1.43;1.67)	-14%	0.001
-1251	**rs62292785**	GG	298	9.73 (9.39;10.08)			4.99 (4.82;5.17)			1.75 (1.70;1.80)		
	- promoter	GA	40	8.56 (7.77;9.43)	-12%	0.016	4.90 (4.45;5.40)	-2%	0.723	1.64 (1.52;1.77)	-6%	0.129
		AA	4	9.21 (6.78;12.51)	-5%	0.730	5.63 (4.14;7.65)	13%	0.448	1.65 (1.30;2.10)	-5%	0.648
-961	**rs7625133**	AA	293	9.67 (9.33;10.03)			5.00 (4.82;5.18)			1.77 (1.72;1.81)		
	- promoter	AC	46	9.00 (8.22;9.84)	-7%	0.140	4.94 (4.52;5.41)	-1%	0.820	1.59 (1.48;1.70)	-10%	0.005
		C C	3	10.19 (7.14;14.54)	5%	0.775	4.74 (3.32;6.75)	-5%	0.765	1.17 (0.89;1.54)	-34%	0.004
-319	**rs193149924**	CC	337	9.58 (9.27;9.91)			4.99 (4.83;5.16)			1.74 (1.70-1.79)		
9	**rs72549254**	GG	247	9.71 (9.34;10.10)			4.89 (4.71;5.08)			1.77 (1.72;1.83)		
	- intron 1	AG	89	9.14 (8.56;9.76)	-6%	0.118	5.34 (5.00;5.69)	9%	0.023	1.66 (1.57;1.74)	-7%	0.023
		AA	6	9.77 (7.60;12.55)	1%	0.965	4.52 (3.53;5.79)	-8%	0.533	1.25 (1.03;1.52)	-29%	0.001
44153	**rs3774275**	AA	164	9.09 (8.67;9.53)			5.34 (5.10;5.59)			1.58 (1.53;1.64)		
	- intron 8	AG	139	10.00 (9.50;10.52)	10%	0.008	4.92 (4.68;5.17)	-8%	0.016	1.84 (1.77;1.91)	16%	<0.001
		GG	39	10.28 (9.33;11.32)	13%	0.025	3.95 (3.60;4.33)	-26%	<0.001	2.04 (1.90;2.19)	29%	<0.001
44259	**rs113938200**	C C	341	9.60 (9.28;9.92)			4.99 (4.83;5.16)			1.74 (1.69;1.78)		
	- exon 9	C T	2	7.40 (4.79;11.43)	-23%	0.242	4.66 (3.02;7.20)	-6%	0.762	1.06 (0.76;1.49)	-39%	0.005
45121	**rs698090**	TT	137	9.14 (8.68;9.63)			5.33 (5.07;5.61)			1.59 (1.53;1.65)		
	-3-UTR	C T	156	9.81 (9.34;10.31)	7%	0.052	5.01 (4.78;5.25)	-6%	0.074	1.80 (1.73;1.87)	13%	<0.001
	MAp44	C C	50	10.12 (9.29;11.02)	11%	0.048	4.10 (3.77;4.45)	-23%	<0.001	1.95 (1.83;2.09)	23%	<0.001
47837	**rs72549257**	AA	313	9.56 (9.24;9.90)			4.98 (4.81;5.16)			1.73 (1.68;1.78)		
	- intron 9	AC	27	9.69 (8.60;10.91)	1%	0.836	5.05 (4.48;5.68)	1%	0.842	1.72 (1.57;1.89)	-1%	0.894
50126	**rs28945068**	C C	316	9.66 (9.33;10.00)			4.98 (4.81;5.15)			1.74 (1.69;1.79)		
	- exon 11	C T	24	8.91 (7.86;10.10)	-8%	0.224	4.90 (4.33;5.56)	-2%	0.812	1.63 (1.47;1.79)	-7%	0.194
55489	**rs72549154**	GG	318	9.69 (9.36;10.02)			4.94 (4.78;5.11)			1.74 (1.70;1.79)		
	- exon 12	GT	25	8.34 (7.38;9.42)	-14%	0.021	5.61 (4.96;6.33)	13%	0.050	1.60 (1.45;1.76)	-8%	0.083
56100	**rs67143992**	GG	228	9.20 (8.84;9.57)			5.33 (5.13;5.54)			1.67 (1.62;1.73)		
	-3-UTR	GA	93	10.29 (9.66;10.95)	12%	0.003	4.50 (4.24;4.77)	-16%	<0.001	1.83 (1.74;1.92)	9%	0.003
	MASP-3	AA	20	10.57 (9.23;12.10)	15%	0.054	3.68 (3.24;4.19)	-31%	<0.001	1.99 (1.79;2.22)	19%	0.002

Δ% indicates the relative change compared to the homozygote state of the major allele. Concentrations are in µg/ml, with 95% confidence intervals. (Pos.) position, (Gen.) genotype, (n) number. Specification of gene-accession number in which the non-synonymous mutations are present; Asp368Asn by rs113938200 in NM_001031849.2 (=MAp44), Gly426Glu by rs28945068 in both NM_001879.5 (=MASP-1) and NM_139125.3 (=MASP-3), Arg576Met by rs72549154 in NM_139125.3 (=MASP-3).

Because of the combined effect of genotypes on the concentration, genotypes were included one-by-one as dependent variables in a linear regression analysis. The data presented in Table 2 concerning MASP-3 and the SNPs were uncorrected for age and gender effects. A model was created where serum concentrations of MASP-3 were age adjusted and gender segregated, resulting in similar results as presented in Table 2 (data not shown). A total of 10 different SNPs showed associations with the concentration of one or more of the three proteins (Table 2). The 10 significant SNPs were evenly distributed with five in the promoter region and five in the MES region of which two were non-synonymous. Six SNPs were associated with MASP-1, five SNPs with MASP-3, and seven SNPs to MAp44 (Table 2).

**Figure 3:**
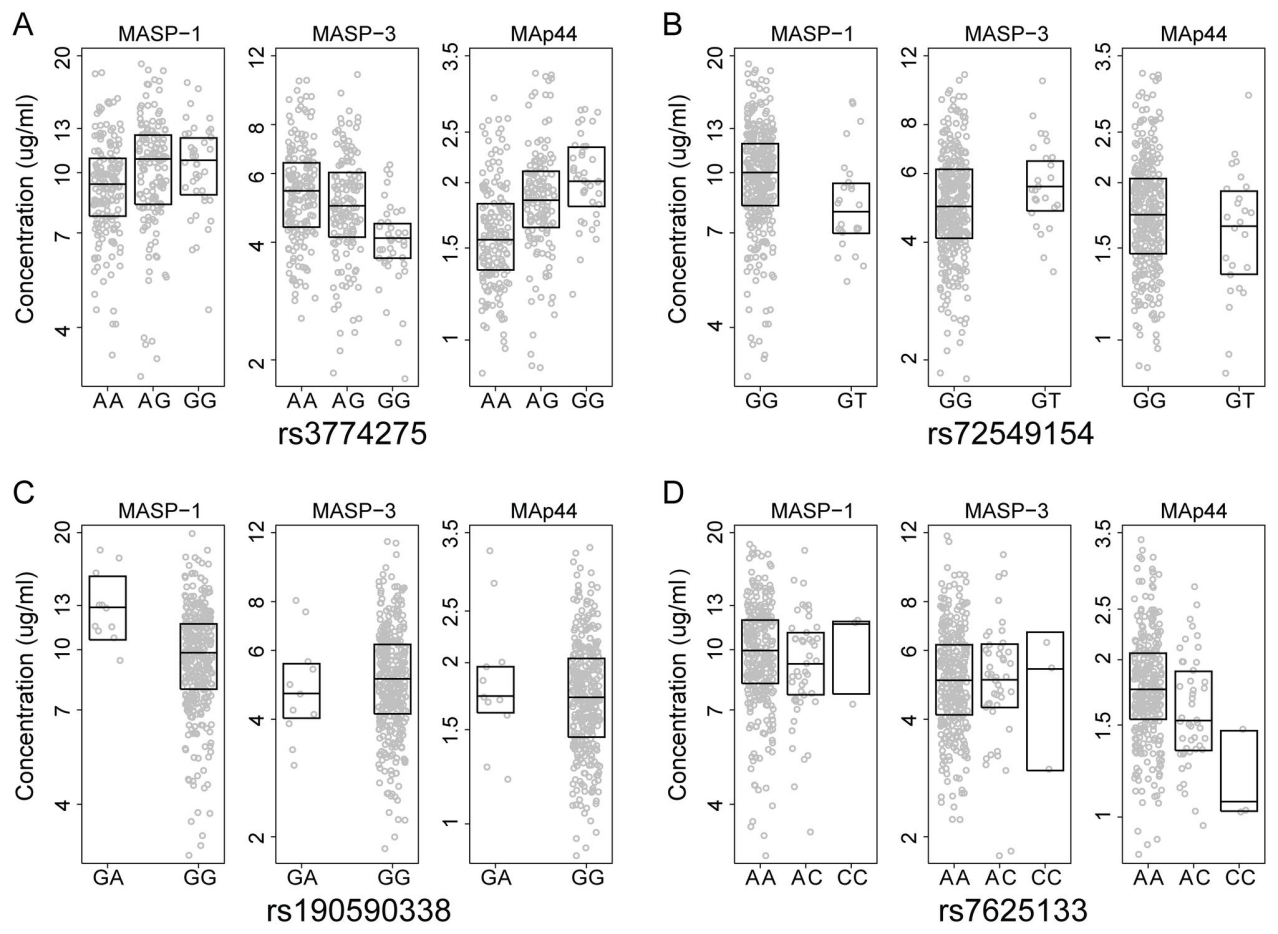
Graphical presentation of the effect of genotypes on MASP-1, MASP-3, and MAp44 concentrations. Each individual is marked with a circle. The box indicates median and inter quartile values. The rs-numbers of the SNPs depicted are given below each graph.

Several of the SNPs had opposite effects on the protein concentrations, i.e. the minor allele of rs3774275 was associated to an allelic dose–response increase in MASP-1 and MAp44 and decrease in MASP-3 (Figure 3A). Being homozygote for the minor allele of rs3774275 was associated with an increase in median concentration of 13% in MASP-1(P = 0.03), 29% in MAp44 (P < 0.001), and a decrease in MASP-3 of 26% (P < 0.001) compared to homozygosis of the major allele. A similar pattern of an allelic dose–response increase in MASP-1 and MAp44 and decrease in MASP-3 was observed for rs698090 and rs67143992. The reverse pattern of allelic dose–response effect was observed for rs72549154 and rs35089177 where presence of the minor allele resulted in an increase of MASP-3 and a decrease of MASP-1 and MAp44 (Figure 3B).

The heterozygote state of rs190590338 had the strongest effect on MASP-1 concentration and lead to an increase of 35% in median concentration when compared to homozygozity of the major allele, while none were homozygotic for the minor allele (Figure 3C). The rs7625133 had the most influence of the promoter variants on the MAp44 concentration, with an allelic dose–response effect of -10% (P = 0.005) when present in heterozygote state and -34% (P = 0.004) when homozygote for the minor allele compared to homozygosis of the major allele (Figure 3D).

### Haplotypes and concentrations

Five haplotypes, H1-H5, were constructed with a frequency ranging from 54 to 7% (Table 3). The global P-values for association between the combined haplotypes and the three *MASP1* related proteins were for MASP-1 0.002, for MASP-3 0.03 and for MAp44 <0.001, proving a significant combined effect of the haplotypes on each of the proteins. The effects of each haplotype were estimated and presented in Table 4 and graphically in Figure S1. The most frequent haplotype, H1, had the highest levels of MASP-1 and MAp44 and lowest levels of MASP-3. The lowest MASP-1 concentration were in the H4 haplotype, with a decrease of 13% (P = 0.002) compared to H1. The H5 haplotype had the highest MASP-3 concentration, increased 16% (P = 0.003) compared to H1. Finally, the lowest MAp44 concentration were in the H3 haplotype, with a decrease of 15% (P = 0.001) compared to H1.

**Table 3:** Haplotypes reconstructed in the *MASP1* gene.

	rs35089177	rs62292785	rs7625133	rs72549254	Frequency (%)
H1	T	G	A	G	54
H2	A	G	A	G	24
H3	T	G	C	A	8
H4	A	A	A	G	7
H5	T	G	A	A	7

Haplotypes were reconstructed by the Haploview software using the “confidence intervals” methods with default settings.

**Table 4:** Estimated geometric mean concentration of the three *MASP1* proteins according to haplotypes.

	**MASP-1**			**MASP-3**			**MAp44**		
	**Concentration**	**Δ%**	**p**	**Concentration**	**Δ%**	**p**	**Concentration**	**Δ%**	**p**
H1	10.50 (9.98;11.04)			4.74 (4.50;4.98)			1.90 (1.83;1.98)		
H2	9.52 (9.02;10.05)	-9%	<0.001	5.01 (4.75;5.29)	6%	0.042	1.74 (1.67;1.81)	-8%	<0.001
H3	9.63 (8.85;10.49)	-8%	0.049	4.78 (4.38;5.20)	1%	0.846	1.62 (1.52;1.73)	-15%	<0.001
H4	9.13 (8.37; 9.97)	-13%	0.002	4.90 (4.48;5.35)	3%	0.457	1.72 (1.61;1.84)	-9%	0.005
H5	9.69 (8.81;10.66)	-8%	0.099	5.49 (4.99;6.04)	16%	0.003	1.75 (1.62;1.88)	-8%	0.026

Effects of haplotypes and P-values are relative to H1. Δ% indicates the relative change compared to H1. Concentrations are in µg/ml, with 95% confidence intervals.

### Non-synonymous SNPs discovered in the *MASP1* gene

We genotyped 346 individuals in the search for three non-synonymous SNPs, and they were present in a total of 48 individuals in heterozygote form with a minor allele frequency ranging from 0.29–3.64% (Table 5). Three individuals carried two non-synonymous SNPs, one carried *p.Asn368Asp* and *p.Arg576Met*, while two individuals carried *p.Gly426Glu* and *p.Arg576Met*. The non-synonymous mutation in exon 9 (rs113938200) causing *p.Asn368Asp* in the unique C-terminal of MAp44, was associated in the heterozygote form with a reduced MAp44 concentration to 61% (P = 0.005), while no effect was observed on the MASP-1 and MASP-3 concentration (P > 0.24). The non-synonymous mutation in exon 11 (rs28945068) causing *p.Gly426Glu* in the protein sequence of MASP-1 and MASP-3 had no influence on the concentration of any of the three proteins in heterozygote form (P > 0.19). The last non-synonymous mutation genotyped was present in exon 12 (rs72549154) causing *p.Arg576Met* in the MASP-3 serine protease. It was found in 25 individuals in heterozygote form, which lead to an increase in MASP-3 concentration of 13% (P = 0.05) and a reduction in MASP-1 of 14% (P = 0.02). Non-synonymous SNPs generally have a high impact on phenotype, and in Table 5 we report the predicted phenotypic effect by four computational tools.

**Table 5:** Predicted effect on protein function of three non-synonymous mutations in *MASP1*.

rs number	Amino acid change	SIFT	Polyphen-2	Grantham score	GERP score	ESP cohort, MAF	Present study, MAF
rs113938200	Asp368Asn	Tolerated	Benign	23	Possibly damaging	0.98%	0.29%
rs28945068	Gly426Glu	Tolerated	Possibly damaging	98	Possibly damaging	1.44%	3.53%
rs72549154	Arg576Met	Deleterious	Benign	91	Benign	3.33%	3.64%

SIFT (Sorting Tolerant From Intolerant). Grantham scores are designated; conservative (0-50), moderately conservative (51–100), moderately radical (101–150), or radical (≥151). GERP (Genomic Evolutionary Rate Profiling). ESP cohort consists of 4300 European-American individuals (Exome Variant Server, NHLBI GO Exome Sequencing Project (ESP), Seattle, WA. 2012). MAF, minor allele frequency.

### Linkage disequilibrium analyses

**Figure 4:**
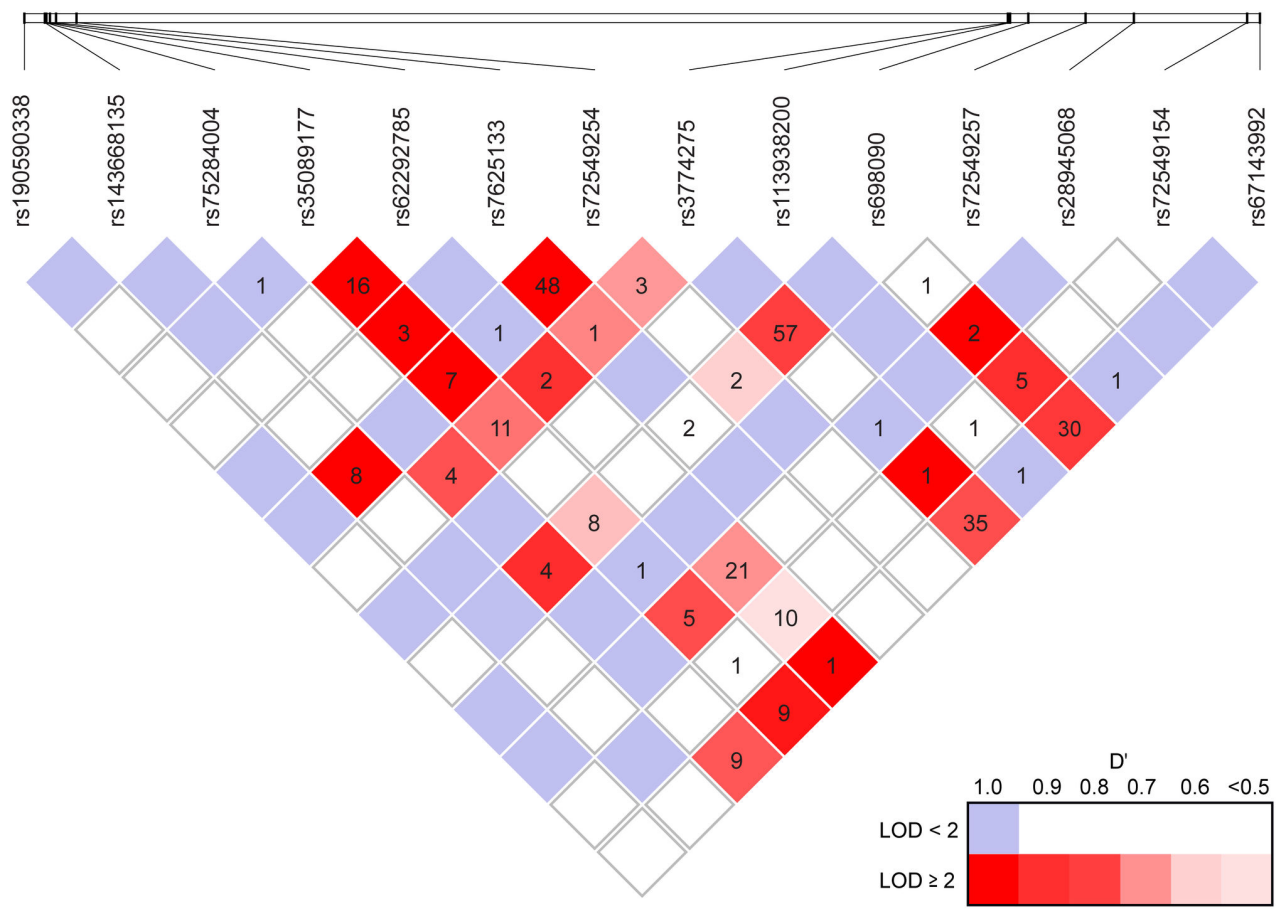
Linkage disequilibrium plot of the polymorphisms investigated in the *MASP1* gene. Numbers in squares are R^2^-values in percent and empty squares indicates R^2^-values <1%. D’ and LOD scores are indicated by a color code.

Linkage analyses revealed that several SNPs were in close linkage as judged by D` = 1, LOD>3, and R^2^-values (Figure 4). Since rs3774275 had a highly significant effect on all three proteins, it was used as a covariate to determine the influence of the remaining 9 SNPs with a significant effect in the multiple linear regression analysis on the serum concentrations of MASP-1, MASP3, and MAp44 (Table 6).

**Table 6:** rs3774275 used as a covariate to determine the influence on the protein concentration of the remaining 9 SNPs.

position	rs-number	MASP-1		MASP-3		MAp44	
		p-value		p-value		p-value	
-2464	rs190590338	0.002	*	0.463		0.451	
-1479	rs75284004	0.699		0.129		0.881	
-1418	rs35089177	0.012	*	0.882		0.260	
-961	rs7625133	0.577		0.447		0.007	*
9	rs72549254	0.470		0.155		0.008	*
44259	rs113938200	0.167		0.802		0.001	*
45121	rs698090	0.983		0.281		0.590	
55489	rs72549154	0.081		0.272		0.705	
56100	rs67143992	0.173		>0.001	*	0.985	

A p-value below 0.05 (*) indicates that the SNP has further explanatory power beyond what is represented by rs3774275.

### MBL, H-ficolin and M-ficolin influence the concentrations of the *MASP1* encoded proteins

**Figure 5:**
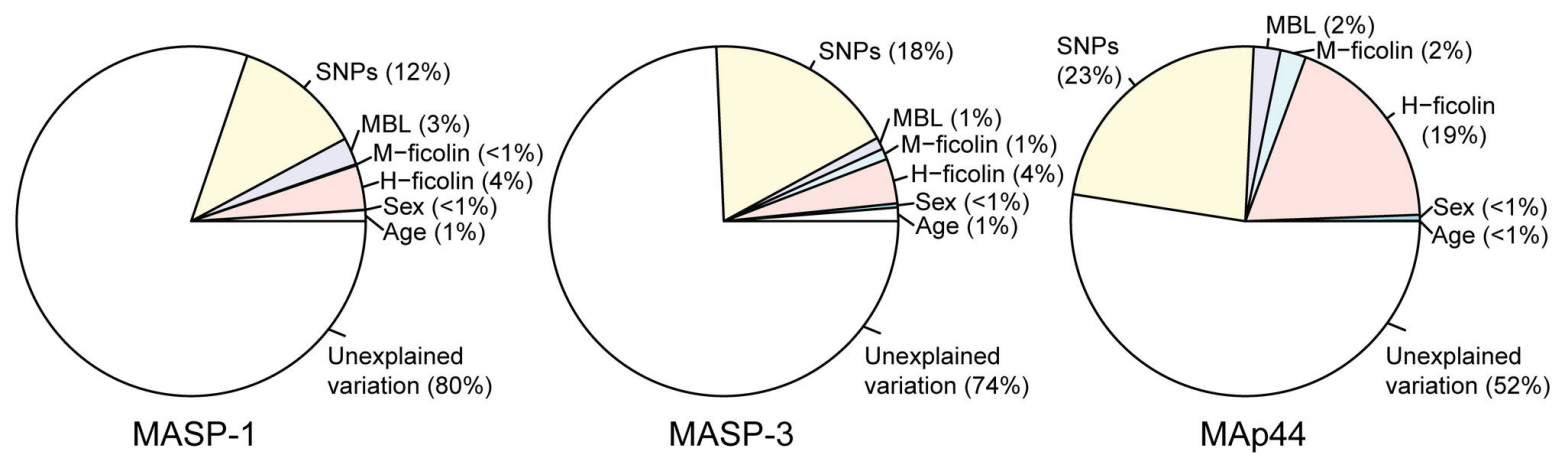
Explained and unexplained variation in the three *MASP1* proteins. The pie charts depict the total amount of variation explained of the three protein concentrations as a result of a multiple regression analysis. “SNPs” represents the total variation of the 15 SNPs investigated.

Since three of the four pattern recognition molecules from the lectin pathway were previously measured in this cohort (MBL [23], H-ficolin [24,25], and M-ficolin [26]), we included the concentrations of these three proteins in a multiple linear regression models with age, gender, and the genetic contribution as independent variables and MAp44, MASP-1, and MASP-3 as dependent variables. As illustrated in Figure 5 this resulted in the explanation of 20% of the total variation for MASP-1, 26% for MASP-3, and 48% for MAp44. The SNPs of the *MASP1* gene contributed with the most explanatory power to all the three proteins, between 12–23%, followed by the concentration of H-ficolin which explained between 4–19% of the variation.

After correction for age, gender, SNPs, MBL, H-, and M-ficolin we finally analyzed whether associations still remained between MAp44, MASP-1, and MASP-3. We calculated the partial correlations of the residuals from a multivariate linear regression model including MAp44, MASP-1, and MASP-3 as outcome variables and age, gender, SNPs, MBL, H-, and M-ficolin as independent variables. There are statistically significant partial correlations between all three proteins, which imply unexplained dependency between the three proteins that does not originate from either of the independent variables. There is a negative partial correlation between MASP-1 and MASP-3(-0.13P = 0.01), positive partial correlations between MASP-1 and MAp44 (0.13P = 0.02), and MAp44 and MASP-3(0.21P < 0.001).

## Discussion

The MES region is a key part of the *MASP1* gene as it is here the mutually exclusive splicing of the primary transcript are done, which generates the three different mRNAs coding for the three *MASP1* proteins. Four SNPs rs3774275, rs698090, rs72549154, and rs67143992 are located within 12 kb of each other in the MES region (Figure 1). The four SNPs have a gene-dose effect on the concentrations of the three proteins, as MASP-1 and MAp44 concentrations increase when MASP-3 decreases and vice versa. Several facts indicate that the four SNPs are markers of the same genetic phenomenon. By regression analysis it was shown that rs698090 and rs72549154 had no further explanatory power besides that of rs3774275, i.e. there was no added value of including either of the SNPs in the model to explain the protein concentration. Indeed rs67143992 had added explanatory power for the concentration of MASP-3 while not of MASP-1 and MAp44, which could reflect that rs67143992 is located in the UTR-region of MASP-3 in exon 12. Furthermore, the LD plot showed that all four SNPs are closely linked. In conclusion, our analysis substantiate that the four SNPs are in strong linkage with a polymorphism that has a substantial effect on the mutually exclusive splicing and thereby influences the protein levels. Unfortunately there is no further explanation for the genetic background for these associations.

Five haplotypes were reconstructed using four SNPs located in the promoter region, which revealed significant associations to concentrations of the three *MASP1* related proteins. The five haplotypes shared similar characteristics of the effects on the proteins, as MASP-1 and MAp44 concentrations increased when MASP-3 decreased and vice versa. So it seems that both at SNP and haplotype levels there are associations between elevated MASP-1 and MAp44 and decreased MASP-3 concentrations.

The only SNP in the MES region that had further explanatory power besides rs3774275 and rs67143992 in the regression analysis was rs113938200. Rs113938200 is a non-synonymous SNP in exon 9 causing *p.Asn368Asp* in the unique C-terminal MAp44, and was not in linkage disequilibrium with any of the other SNPs tested. Three of the four computational methods used to predict the phenotypic effect favored a benign outcome of this mutation (Table 5). Although rs113938200 was associated in the heterozygote form with a reduction of MAp44 concentration to 61%, one should be cautious in the interpretation of this result as the monoclonal-antibody used in the MAp44 assay was specifically raised against the 17 amino acids compromising the unique C-terminal of MAp44 [4]. So the low concentration of MAp44 could result from low affinity of the monoclonal antibody for the altered MAp44 C-terminal peptide.

In the promoter region of *MASP1* several SNPs deserve special attention. The SNP of rs190590338 was located in the outermost part of the promoter region, and was associated with an increase of 35% in MASP-1 concentration in heterozygotic state while MASP-3 and MAp44 concentrations were unaffected. We found no individual homozygotic for the variant, but suspect that such individuals would have even higher MASP-1 levels. An in-silico transcription factor analysis showed that the mutation of rs190590338 lead to gain of function of a binding site for CCAAT enhancer binding protein alpha (C/EBP alpha) (data not shown) [27]. C/EBP alpha is expressed at high levels in liver and adipose tissue, and has many important physiological roles including the regulation of terminal hepatocyte differentiation and function [28]. It is plausible that the isolated effect of rs190590338 on MASP-1 concentration is owing to a transcription factor, which is differentially expressed throughout the body, with predominance for the liver where mRNA for MASP-1 is almost exclusively expressed as opposed to MASP-3 and MAp44 [4]. This hypothesis is further supported as rs190590338 was not in linkage with any of the other SNPs in the promoter or MES region (Figure 4).

Two SNPs in the promoter region (rs7625133) and intron 1 (rs72549254) have effect solely on the MAp44 concentration and they are in very close linkage. The effect of the two SNPs were dose-dependent, and heterozygosity of either SNP lead to a reduction in MAp44 concentration of 7-10%, while homozygosity of either mutation was associated with a major decrease of 29-34% compared to homozygosity of the wildtype. The in-silico transcription factor analysis did not reveal either gain or loss of a transcription binding site at the site of rs7625133 (data not shown) [27]. It is likely that the genetic effect reflected by the two SNPs on MAp44 concentration is caused by a transcription factor, which is differentially expressed, with predominance for the heart where MAp44 is highly expressed as the only one of the lectin pathway proteins [4]. Whether MAp44 is associated with cardiovascular diseases in anyway is unknown, but both rs7625133 and rs72549254 would be relevant SNPs to investigate in future genetic studies regarding cardiovascular disease.

The three non-synonymous SNPs investigated in the study, are also the most frequent ones out of 57 SNPs (non-synonymous, frame shift, and stop mutations) reported in 4300 European-Americans in *MASP1* in the Exome Variant Server [29]. All three SNPs have a reported minor allele frequency (MAF) above 1% in the ESP cohort, with rs28945070 (*Gly510Ser* in MASP-1) in fourth place having a MAF = 0.22% and the other 53 SNPs were very rare with MAF ≤ 0.06%. The MASP1 gene is well conserved clearly indicating that the three MASP1 proteins possess important physiological functions. This is further substantiated by rare mutations in the MASP-3 encoding part of the MASP1 gene have been linked to the rare autosomal recessive congenital syndrome 3MC [11,12], and that lack of MASP-1 due to genetic mutations leads to loss of the complement activating by the lectin pathway [6].

Currently we do not know whether higher levels of circulating MASP-1 have a clinical effect in either health or disease. With the recent advances made by Degn et al., demonstrating that MASP-1 is crucial for the lectin pathway through the activation of MASP-2 [6], it is plausible that an increase in MASP-1 concentration of 35% could be clinically important. This could lead to a more readily activated lectin pathway with increased levels of inflammation. Such a scenario could be wanted when facing pathogenic microorganisms thereby facilitating their clearance, but would be inappropriate in an autoimmune response where it would augment the secondary damage to tissues. More studies are needed where both serum levels and relevant polymorphisms are investigated in different patient populations.

It is known that the production of the components of the C1 complex of the classical complement activation pathway is regulated so that equimolar amounts of C1q, C1r2, and C1s2 are found in circulation even though these molecules have different tissue origins [30]. We thus speculated that the concentration of the pattern recognition molecules from the lectin pathway could influence the concentrations of the three *MASP1* proteins, as they are circulating in plasma together in complexes. H-ficolin explained 4-19% of the variation whereas MBL and M-ficolin only explained 1-3% of the variation in the *MASP1* related proteins. We speculate that with H-ficolin being present at a 13 times higher molecular concentration (52 nM) than MBL (4 nM) and M-ficolin (4 nM) [19], this would allow more *MASP1* related proteins to bind to H-ficolin and thereby less prone to degradation in serum leading to a higher concentration.

We further investigated for additional correlations between the three *MASP1* proteins not explained by the independent variables. There were significant negative partial correlations between the residuals of MASP-1 and MASP-3 and positive partial correlations between MASP-1 versus MAp44 and MASP-3 versus MAp44. This implicates that there exist other unknown factors that influence the protein concentrations. A straightforward explanation for these correlations could be genetic contributions that were unexplained by this study. The 15 SNPs investigated in this study spans combined 15kb of the promoter and MES region while the *MASP1* gene covers 76kb of exon and intron regions, leaving large parts of the *MASP1* gene concealed.

MASP-3 concentrations were associated with age, gender, and their interaction, but we are cautious about the interpretation of such finding as neither MASP-1 nor MAp44 showed similar associations, and there are no apparent biological explanations that could render such an association probable. It is further documented that age and gender had minimal influence as their combined explanatory power of the variance in a multivariate regression model was less than 2%.

The major strengths of the study was the use of exploratory sequencing of a minor group of blood donors with extreme values of the three *MASP1* proteins, which increased the chance of finding genetic variants with a substantial impact on protein concentrations. A weakness of the study is that the sequencing analyses were performed on a minor part of the exons, and that the MES region was left unexplored, which was due to financial constraints.

The observed associations between genotypes and the three *MASP1* proteins were found in healthy individuals. It remains to be seen whether differential expression would be observed in individuals during acute phase reaction or various disease processes, either of which might lead to altered transcription. The present study generated new knowledge through interlinking genotype and phenotype of MASP-1, MASP-3, and MAp44 and the *MASP1* gene opening up for future genetic studies of the innate immune system in health and disease.

## Supporting Information

Figure S1 **Figure S1.**
(TIF)

Table S1 **SNPs exploration sequencing in MASP1 in 46 individuals.** All SNPs were in Hardy-Weinberg equilibrium except rs72549284, which had an observed heterozygosity of 0, a predicted heterozygosity of 0.124 and a Hardy-Weinberg equilibrium p value =0.002. This was most likely due to only 71.4% were genotype for this SNP. SNPs in bold were investigated further in 350 individuals.
(DOCX)

Table S2 **Assay information for the 15 SNPs genotyped in 346 blood donors.** Data on the forward and reverse primers regarding the not custom-designed assays are not available due to commercial reasons.
(DOCX)
