# Supplementary figures and images for: Polymorphisms in the MASP1 Gene Are Associated with Serum Levels of MASP-1, MASP-3, and MAp44
Source: PLoS One. 2013 Sep 2;8(9):e73317. doi: 10.1371/journal.pone.0073317 (PMC3759447; doi:10.1371/journal.pone.0073317)

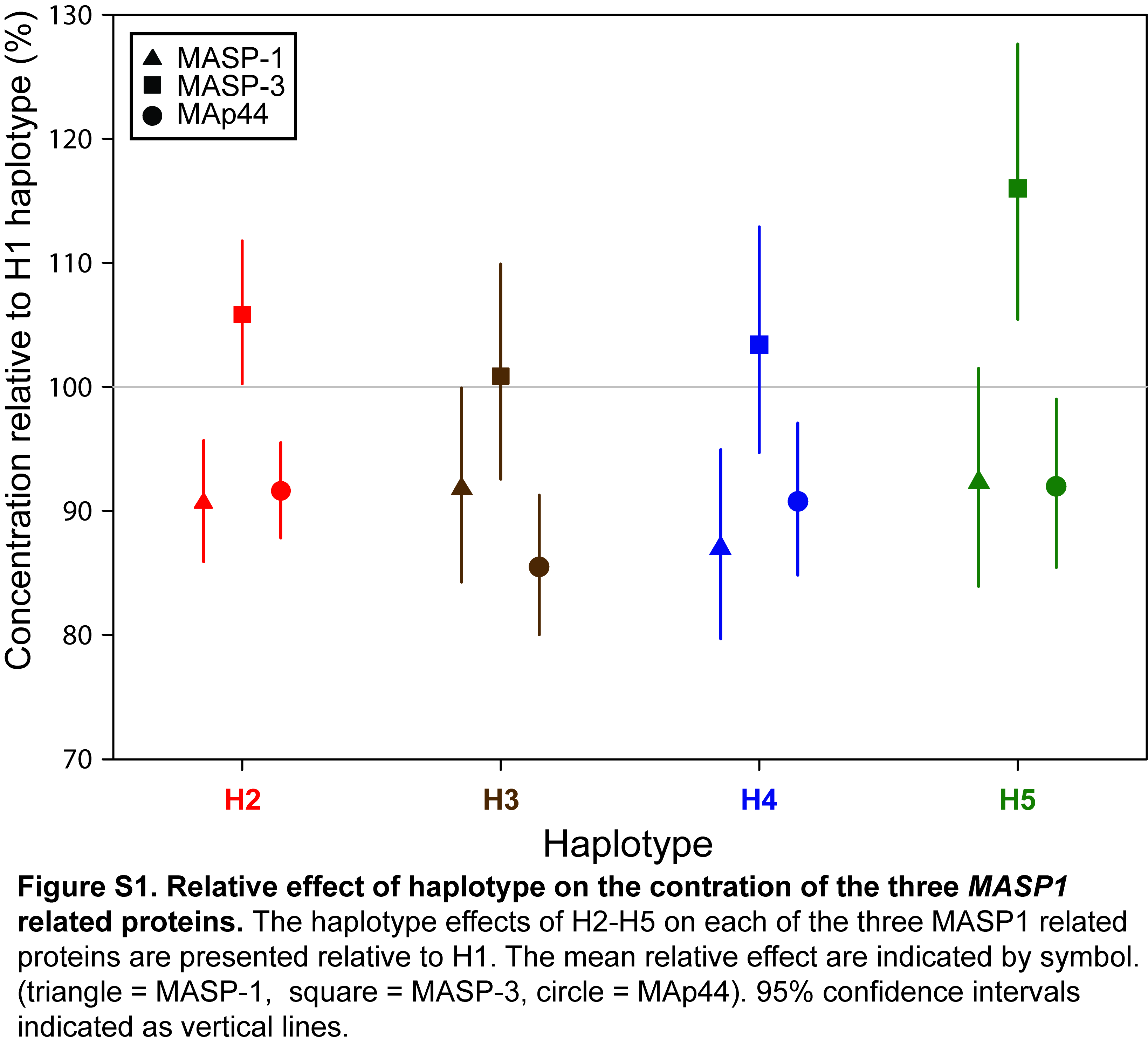

Supplement: Figure S1 — (TIF) [file pone.0073317.s001.tif]
