# Supplementary material for: Polymorphisms in the MASP1 Gene Are Associated with Serum Levels of MASP-1, MASP-3, and MAp44
Source: PLoS One. 2013 Sep 2;8(9):e73317. doi: 10.1371/journal.pone.0073317 (PMC3759447; doi:10.1371/journal.pone.0073317)
Supplement: Table S1 — SNPs exploration sequencing in MASP1 in 46 individuals. All SNPs were in Hardy-Weinberg equilibrium except rs72549284, which had an observed heterozygosity of 0, a predicted heterozygosity of 0.124 and a Hardy-Weinberg equilibrium p value =0.002. This was most likely due to only 71.4% were genotype for this SNP. SNPs in bold were investigated further in 350 individuals. (DOCX) [file pone.0073317.s002.docx]

| **Region** | **rs id** | **Chr placering** | **Upstream sequence** | **Allels** | **Downstream sequence** | **Aminoacid change** | **% Genotyped** | **Minor allele frequency** |
| --- | --- | --- | --- | --- | --- | --- | --- | --- |
| promoter | **rs190590338** | 187,011,884 | CATATCAGAT | C:T | TGCTATATAT |  | 100 | 0.012 |
| promoter | **rs143668135** | 187,010,915 | TATGAATGCC | G:A | TCTGTGTGTG |  | 100 | 0.012 |
| promoter | **rs75284004** | 187,010,899 | GTGTGTGCAT | T:C | TGAAATTCCA |  | 100 | 0.048 |
| promoter | **rs35089177** | 187,010,838 | TGGCTAACAC | T:A | TCAAATTCTG |  | 100 | 0.321 |
| promoter | **rs62292785** | 187,010,671 | TGCCTGACAC | G:A | TAGCACTCAA |  | 100 | 0.107 |
| promoter | **rs7625133** | 187,010,381 | AATTTTCTTA | T:G | TATTCAGCTC |  | 97.6 | 0.122 |
| promoter | rs13089330 | 187,010,270 | TTCTTGCTGC | G:A | ATGGGCCCTT |  | 97.6 | 0.317 |
| promoter | rs7624953 | 187,010,159 | ACTTGGTGCT | T:C | CCCCAGTTCC |  | 97.6 | 0.195 |
| promoter | ss748770621 | 187,010,134 | CCGTGGCTCT | G:T | TTCACAGGAA |  | 100 | 0.012 |
| 5´-UTR | rs72549284 | 187,010,082 | GAAAACCTGT | A:C | GTTATTTGGG |  | 71.4 | 0.067 |
| 5´-UTR | ss748770622 | 187,009,803 | CACACACACA | G:C | AGTGATACAA |  | 100 | 0.19 |
| 5´-UTR | rs74342018 | 187,009,760 | TATTTTCTCT | C:G | AAGGGCTGAA |  | 100 | 0.19 |
| 5´-UTR | rs75502847 | 187,009,742 | GAAGTCAGCC | A:G | CACAGGATAA |  | 100 | 0.19 |
| 5´-UTR | **rs193149924** | 187,009,739 | GTCAGCCACA | C:T | AGGATAAAGG |  | 100 | 0.048 |
| 5´-UTR | rs78795981 | 187,009,738 | TCAGCCACAC | A:G | GGATAAAGGA |  | 100 | 0.19 |
| intron 1 | **rs72549254** | 187,009,412 | AAATGAGGTA | C:T | GTGCCAGGGG |  | 100 | 0.19 |
| intron 1 | rs16861896 | 187,009,372 | TTTTGGGGAC | C:T | AGTGTCAAAT |  | 100 | 0.19 |
| intron 1 | rs16861895 | 187,009,351 | AGCAGGGAAG | G:C | TCTGACCTTG |  | 100 | 0.19 |
| Intron 2 | rs698105 | 187,003,495 | AACATCACAA | C:T | GGTATGAATT |  | 97.6 | 0.195 |
