# Supplementary material for: Polymorphisms in the MASP1 Gene Are Associated with Serum Levels of MASP-1, MASP-3, and MAp44
Source: PLoS One. 2013 Sep 2;8(9):e73317. doi: 10.1371/journal.pone.0073317 (PMC3759447; doi:10.1371/journal.pone.0073317)
Supplement: Table S2 — Assay information for the 15 SNPs genotyped in 346 blood donors. Data on the forward and reverse primers regarding the not custom-designed assays are not available due to commercial reasons. (DOCX) [file pone.0073317.s003.docx]

| **rs id** | **Assay ID** | **Assay technique** | **Forward Primer Sequence** | **Reverse Primer Sequence** | **VIC labelled probe** | **FAM labelled probe** |
| --- | --- | --- | --- | --- | --- | --- |
| rs190590338 | Custom designed | TaqMan OpenArray | GGGCCACGTAGTTCCTGTTT | GGCTGCCAATGTTCAATGGAAAG | CCAATATATAGCAgATCTGA | CCAATATATAGCAaATCTGA |
| rs143668135 | Custom designed | TaqMan OpenArray | GGGTAGAAAGGAAAGATTACAGCTAAACT | GCCACTGTATTACACTGCTACTCA | ACACAGAcGGCATTC | ACACAGAtGGCATTC |
| rs75284004 | Custom designed | TaqMan OpenArray | GAGTTTCTCTATGAATGCCGTCTGT | CTTTCCCAAGTTCACACAGCTAGAA | ATGGAATTTCAaATGCAC | ATGGAATTTCAgATGCAC |
| rs35089177 | Custom designed | TaqMan OpenArray | CTTTCCCAAGTTCACACAGCTAGA | GTCTGTGTGTGTGCATTTGAAATTC | AGTGGCTAACACtTCAAAT | TGGCTAACACaTCAAAT |
| rs62292785 | Custom designed | TaqMan OpenArray | CTGCCACTAGCACTAGAAGCT | CTCTCTGGGCAATTTCCTCTGA | CCTGACACgTAGCACT | CCTGACACaTAGCACT |
| rs7625133 | C__29113675_10 | TaqMan OpenArray |  |  |  |  |
| rs193149924 | Custom designed | TaqMan OpenArray | GAGCCCCTCAGTTATTTTCTCTCAA | CTCCCAGGAACCTGACAACA | AGCCACAcAGGATAA | TCAGCCACAtAGGATAA |
| rs72549254 | Custom designed | Single TaqMan assay | TGGGACCCCACGTTATGC | ACTGTCACATTGGGATTGGAGATG | CTGCCAAgGCTTGAG | CTGCCAAtGCTTGAG |
| rs3774275 | C___3289662_10 | TaqMan OpenArray |  |  |  |  |
| rs113938200 | Custom designed | TaqMan OpenArray | CCCGTCATTCACTCTGTCACTTG | TCTCTCTGCTTCCTTCCTCTGTATC | TCTCCAGATcGATTTC | TCTCCAGATtGATTTC |
| rs698090 | C____834837_10 | TaqMan OpenArray |  |  |  |  |
| rs72549257 | Custom designed | TaqMan OpenArray | ATGAATGGAAGAGACCTCAAAAGCA | GCACACAATTCCAGAAAGCAATGTT | CATGGGTaGCTTCAC | ATGGGTcGCTTCAC |
| rs28945068 | C__58141621_10 | TaqMan OpenArray |  |  |  |  |
| rs72549154 | Custom designed | TaqMan OpenArray | TGGGACCCCACGTTATGC | ACTGTCACATTGGGATTGGAGATG | CTGCCAAgGCTTGAG | CTGCCAAtGCTTGAG |
| rs67143992 | Custom designed | TaqMan OpenArray | GAGCCTTTTCCCTATACCACACT | CCTAGCGGTGGCTTCTCCTA | ACCCTGAgAGGCAG | ACCCTGAaAGGCAG |
